# Individual Constituents from Essential Oils Inhibit Biofilm Mass Production by Multi-Drug Resistant *Staphylococcus aureus*

**DOI:** 10.3390/molecules200611357

**Published:** 2015-06-19

**Authors:** Laura Espina, Rafael Pagán, Daniel López, Diego García-Gonzalo

**Affiliations:** 1 Tecnología de los Alimentos, Departamento de Producción Animal y Ciencia de los Alimentos, Facultad de Veterinaria, Universidad de Zaragoza, Zaragoza 50013, Spain; E-Mails: espina@unizar.es (L.E.); pagan@unizar.es (R.P.); 2 Instituto Agroalimentario de Aragón (IA2), Universidad de Zaragoza-CITA, Zaragoza 50013, Spain; 3 Research Centre for Infectious Diseases (ZINF), University of Würzburg, Würzburg 97080, Germany; E-Mail: daniel.lopez@uni-wuerzburg.de

**Keywords:** biofilms, *Staphylococcus aureus*, carvacrol, citral, (+)-limonene, essential oils

## Abstract

Biofilm formation by *Staphylococcus aureus* represents a problem in both the medical field and the food industry, because the biofilm structure provides protection to embedded cells and it strongly attaches to surfaces. This circumstance is leading to many research programs seeking new alternatives to control biofilm formation by this pathogen. In this study we show that a potent inhibition of biofilm mass production can be achieved in community-associated methicillin-resistant *S. aureus* (CA-MRSA) and methicillin-sensitive strains using plant compounds, such as individual constituents (ICs) of essential oils (carvacrol, citral, and (+)-limonene). The Crystal Violet staining technique was used to evaluate biofilm mass formation during 40 h of incubation. Carvacrol is the most effective IC, abrogating biofilm formation in all strains tested, while CA-MRSA was the most sensitive phenotype to any of the ICs tested. Inhibition of planktonic cells by ICs during initial growth stages could partially explain the inhibition of biofilm formation. Overall, our results show the potential of EOs to prevent biofilm formation, especially in strains that exhibit resistance to other antimicrobials. As these compounds are food additives generally recognized as safe, their anti-biofilm properties may lead to important new applications, such as sanitizers, in the food industry or in clinical settings.

## 1. Introduction

Microbial communities adhered to surfaces are commonly referred as biofilms and are well recognized to cause hard-to-treat bacterial infections, being associated with around 65% of all human bacterial infections worldwide and with 80% of persistent infections in the United States [1,2,3]. The human pathogen *Staphylococcus aureus* shows a great ability to form biofilms, which represent a serious threat to the food industry and clinical settings [4]. *S. aureus* is well adapted to human colonization because it is part of the normal microbiota of the nasopharynx and skin, however, *S. aureus* penetrates to the internal parts of the body during surgical practice or food consumption and causes severe infections that can progress to life-threatening diseases, like necrotizing fasciitis, endocarditis, or pneumonia [4]. In *S. aureus* biofilms, cells are embedded in a self-produced extracellular matrix that protects cells not only from the host’s immune response but also from any antimicrobial treatment [5].

Bacterial biofilms are particularly challenging in the food industry because bacteria contaminate food surfaces (e.g., produce or animal carcasses) or food-contact surfaces (e.g., equipment and processing environment), and once biofilm is formed, its eradication becomes difficult because it exhibits great resilience to environmental stresses, disinfectants, and antimicrobial treatments [6]. As a consequence, these biofilms can act as reservoirs of persistent contaminations, cross-contaminations, and post-processing contaminations, leading to food spoilage with a potential risk to public health [2,7]. Several factors can account for the contamination of food products with *S. aureus* that often occurs during handling and packaging in food industries, including its frequent occurrence on food contact surfaces and the ability of some strains to form biofilms in food equipment and environments [8], and its common presence as a commensal colonizer of the skin and mucous membranes of healthy animals and humans [3,7].

Prevention of biofilm formation is considered preferable to its removal, since the latter is a very difficult and demanding task, which can cause recontamination problems due to release of bacterial cells and toxins after disruption of the biofilms [9]. Thus, prevention of biofilm formation of *S. aureus* in food and clinical environments is required in order to minimize the potential risk of a widespread dissemination of these new multi-drug resistant strains [7,10]. Control measures mostly rely on the application of effective cleaning and disinfecting procedures [11,12]. Over the last years, the proven antimicrobial efficacy of plant-derived essential oils (EOs) and their individual constituents (ICs) [13] has elicited their proposal as a “green alternative” to the routinely used disinfecting products such as sodium hypochlorite or quaternary ammonium compounds [14]. Many EOs and ICs are incorporated in disinfectant compositions and proposed as novel food preservatives [15] mainly due to their strong activity in the inhibition and inactivation of bacteria and fungi. However, more research is needed in order to have a better approach to develop the potential use of EOs and ICs as inhibitors of the biofilm formation by *S. aureus* in food and clinical environments. For example, inhibition of biofilm formation by these compounds is usually evaluated after 24 h of treatment [16,17], without considering that biofilm formation is a dynamic and cyclical process involving attachment, maturation and a final dispersal phase [18]. Due to strong bacteriostatic and bactericidal activity shown by the ICs (+)-limonene, citral, and carvacrol against planktonic cells, such as *S. aureus*, *Listeria monocytogenes*, *Escherichia coli*, *Enterococcus faecium*, *Salmonella enterica*, or *Pseudomonas aeruginosa* [19,20,21], we selected these ICs to evaluate their potential as inhibitors of biofilm mass production by *S. aureus* strains. In order to cover different types of strains, two community-associated methicillin-resistant *S. aureus* (CA-MRSA) strains (SC-01 and USA300) and two methicillin-sensitive *S. aureus* (MSSA) strains (Newman and UAMS-1) were chosen. The main objective of this work was to study the effect of different concentrations of carvacrol, citral, or (+)-limonene on the ability of clinical isolates of *S. aureus* to form biofilms *in vitro*. Additionally, the effects of these compounds on planktonic cells and their implication in the inhibition of biofilm mass production were studied.

## 2. Results and Discussion

### 2.1. Selection of Effective Compounds According to Their MICs

The potential activity of ICs as inhibitors of biofilm mass production was firstly addressed by determining the MIC of each compound in *S. aureus* planktonic cells (Table 1). Interestingly, MICs of ICs were similar against all *S. aureus* strains. Thus, (+)-limonene showed a MIC of 5000 µL/L against all bacterial strains assayed. For all the strains, the MICs for carvacrol and citral were lower than for (+)-limonene (200 and 500 µL/L, respectively). Therefore, non-inhibitory concentrations (NICs) were determined at 100, 200, and 2000 µL/L for carvacrol, citral, and (+)-limonene, respectively, against all strains tested.

**Table 1:** MICs of ICs against *S. aureus* strains. Minimum inhibitory concentrations (MICs) in µL/L of individual constituents (carvacrol, citral, or (+)-limonene) against *Staphylococcus aureus* strains (SC-01, USA300, UAMS-1, and Newman). The non-inhibitory concentration (NIC) is shown in parentheses.

	SC-01	USA300	UAMS-1	Newman
**Carvacrol**	200 (100)	200 (100)	200 (100)	200 (100)
**Citral**	500 (200)	500 (200)	500 (200)	500 (200)
**(+)-Limonene**	5000 (2000)	5000 (2000)	5000 (2000)	5000 (2000)

### 2.2. Biofilm Development throughout Time of S. aureus Strains in the Absence and the Presence of ICs

**Figure 1:**
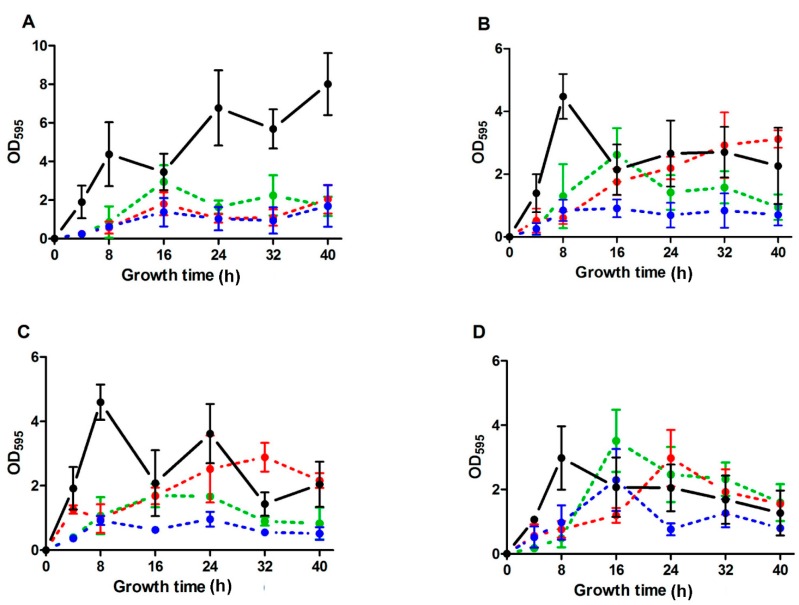
Inhibition of biofilm mass production in presence of NIC of carvacrol, citral and (+)-limonene. Biofilm development (expressed as optical absorbance units at 595 nm) at 37 °C of *Staphylococcus aureus* SC-01 (**A**); USA300 (**B**); UAMS-1 (**C**); or Newman (**D**) in the absence of antimicrobial compounds (●) or in the presence of the non-inhibitory concentration (NIC) of each compound: 100 µL/L of carvacrol (●), 200 µL/L of citral (●), or 2000 µL/L of (+)-limonene (●).

As a preliminary step to evaluate activity of antimicrobial compounds against biofilm formation, we quantified biofilm mass production by all strains. Thus, the biofilm mass was measured during 40 h of incubation (Figure 1).

Using this approach, the patterns of biofilm formation and biofilm thickness were evaluated for each strain at every time point. These patterns were characterized by the alternation of the assembly-dissasembly of the biofilm. CA-MRSA strain, SC-01 formed the thickest biofilm as measured at any growth time from 16 h onwards, reaching a biofilm mass corresponding to 8 absorbance units (AU) after 40 h (Figure 1A).

Besides, it presented a different growth pattern from the other three strains, since it continued increasing its biofilm mass throughout the whole experiment. In contrast, the biofilms formed by UAMS-1 (MSSA) and USA300 (CA-MRSA) increased throughout the first 8 h of incubation (over 4 AU) and then biofilm mass decreased (Figure 1B,C). Moreover, Newman strain (MSSA) presented the weakest biofilm forming ability: a growth plateau or declining phase started after peaking at 8 h, and the measured absorbance values remained under 3 AU (Figure 1D).

The effect of the addition of the NIC of each IC on the biofilm development was evaluated by the representation of the absorbance values throughout time (Figure 1). The most prominent effect observed was the decrease in the biofilm mass after 8 h of incubation in the presence of all ICs tested, which corresponded to the peak time point of biofilm mass production in the Newman and UAMS-1 strains (MSSA), and the USA300 strain (CA-MRSA). In addition to this, we detected an important reduction in the production of biofilm mass in *S. aureus* SC-01 after 8 h onwards of incubation time in the presence of any of the ICs in the growth medium (Figure 1A) (*p* < 0.05). *S. aureus* Newman was the least susceptible strain to the action of the ICs, since their addition seemed to cause a delay in the biofilm development rather than a reduction in the biofilm mass (Figure 1D). Yet, Newman strain formed weak biofilms in all conditions tested, which prevented us from characterizing with precision the efficiency of the ICs inhibiting biofilm formation in this particular strain.

Although we detected similar anti-biofilm activity of the three ICs at their respective NICs, carvacrol was active at a lower concentration. Carvacrol decreases the biofilm mass production of USA300 and UAMS-1 at every time point tested (Figure 1B,C). This contrasted to the anti-biofilm activity of the rest ICs, which showed an inhibitory effect that was dependable of the incubation period. For instance, the addition of 2000 µL/L of (+)-limonene reduced the production of biofilm mass in *S. aureus* USA300 by 90% after 8 h of incubation, but increased it by 30% after 40 h of incubation (Figure 1B). As a consequence, to compare the effect of each IC on *S. aureus* strains in relation to the differences in biofilm development between strains and throughout time, we evaluated the decrease in the total biofilm mass production (expressed as the cumulative absorbance thickness measured over 40 h of incubation period) for each strain when adding each IC in comparison with the control (Table 2).

**Table 2:** Global inhibitory effect of ICs in biofilm formation by *S. aureus*. Percentage of decrease in the cumulative absorbance values in the presence of non-inhibitory concentration (NIC) of carvacrol, citral, or (+)-limonene against *Staphylococcus aureus* strains (SC-01, USA300, UAMS-1, and Newman), in comparison to each value in the absence of added compounds (control). Cumulative absorbance values indicating the biofilm thickness were measured throughout time for a total of 40 h. Values were obtained from the measurement of the area under the curve (AUC) and are expressed in optical absorbance units (at 595 nm)·h (mean ± standard deviation). Asterisks indicate statistically significant differences between each mean AUC value in the presence of each compound with that of the AUC value in its absence. Different superscript letters indicate statistically significant differences in the percentage of decrease in the AUC value among strains for the same compound. Different superscript numbers indicate statistically significant differences in the AUC values among compounds for the same strain. ANOVA tests with Bonferroni’s multiple comparison Post-tests were used (α = 0.05).

	SC-01	USA300	UAMS-1	Newman
Carvacrol	81.51 *^,a1^ ± 11.16	74.62 *^,a1^ ± 12.23	74.66 *^,a1^ ± 2.79	33.28 *^,b1^ ± 17.34
Citral	65.00 *^,a1^ ± 15.30	47.34 *^,a1,2^ ± 21.50	57.10 *^,a2^ ± 6.75	0.21 ^b1^ ± 29.78
(+)-Limonene	77.77 *^,a1^ ± 6.06	28.54 ^b2^ ± 19.45	26.46 ^b2^ ± 11.83	11.19 ^b1^ ± 9.42

Thus, Table 2 shows that SC-01 was the most sensitive strain to any IC, decreasing its total biofilm mass production to at least 65%; and that Newman experienced a significantly lower reduction in its biofilm production when compared to the other strains, regardless of the added compound (*p* < 0.05). Carvacrol was the most effective IC regardless of the tested strain, decreasing more than 30% of biofilm mass production to all strains tested (Table 2) and being statistically more effective than (+)-limonene (*p* < 0.05).

**Figure 2:**
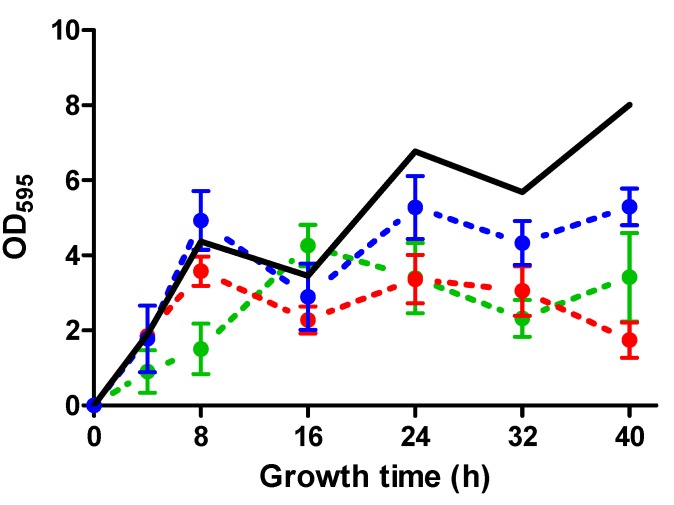
Inhibition of biofilm mass production by *S. aureus* SC-01 in presence of 0.1× NIC of carvacrol, citral and (+)-limonene. Biofilm development (expressed as optical absorbance units at 595 nm) at 37 °C of *Staphylococcus aureus* SC-01 in the absence of antimicrobial compounds (continuous line) or in the presence of 1/10 the non-inhibitory concentration (NIC) of each compound: 10 µL/L of carvacrol (●), 20 µL/L of citral (●), or 200 µL/L of (+)-limonene (●).

Next, we further evaluated the potential of ICs at lower concentrations to inhibit biofilm mass production in the SC-01 strain (CA-MRSA). Figure 2 was obtained by evaluating the evolution of the biofilm mass of SC-01 in the presence of 0.1-fold NIC of each IC. These conditions evidenced a reduction in the biofilm mass by approximately 40%, 55%, and 80% after 40 h of incubation using ICs concentrations as low as 10, 20, or 200 µL/L of carvacrol, citral, and (+)-limonene, respectively.

### 2.3. Effect of ICs on Planktonic Cells in Relation to Biofilm Formation

The effect of the NIC of ICs on Newman strain would be consistent with a delay in the development of the biofilm formation, differing from the effect of the inhibition of biofilm formation that we observed in SC-01 strain (Figure 1A,D). Based on these results, we hypothesized that ICs could affect the viability of planktonic cells, which may alter the regular cycle of biofilm formation and would explain the differences in the biofilm mass that were observed in the absence and the presence of ICs.

**Figure 3:**
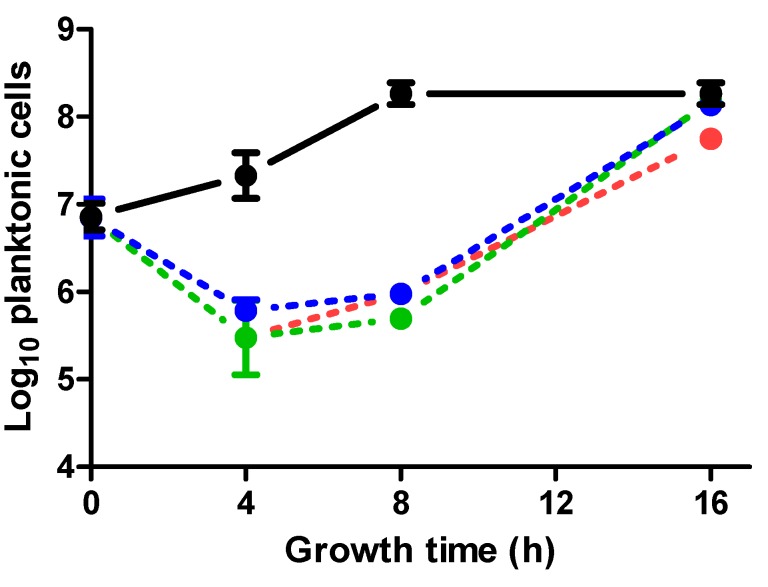
Inactivation of planktonic cells at sub-MIC values of carvacrol, citral, and (+)-limonene. Log_10_ concentration of planktonic cells (CFU/mL) of *Staphylococcus aureus* Newman when grown at 37 °C on polystyrene surfaces in growth medium without added antimicrobial compounds (●), or in the presence of the non-inhibitory concentration (NIC) of each compound: 100 µL/L of carvacrol (●), 200 µL/L of citral (●), or 2000 µL/L of (+)-limonene (●).

This antimicrobial effect of ICs is probably due to the coexistence of bacterial cells exhibiting different physiologies and thus showing different sensitivities to the action of our compounds. In order to evaluate the correlation between the effect of ICs on planktonic cells and the biofilm development, the planktonic cell concentration of *S. aureus* Newman was measured during early stages of biofilm formation. As shown in Figure 3, the concentration of planktonic cells increased from 10^7^ to 10^8^ CFU/mL after the first 8 h of incubation in the absence of antimicrobials. However, in the presence of the NIC of any IC the initial concentration of planktonic cells decreased about 1.5 log_10_ cycles. Similar results were obtained for the other strains (data not shown).

### 2.4. Discussion

Natural compounds with antimicrobial and anti-biofilm activity are being promoted for different purposes, such as food preservatives, disinfectants and chemotherapeutic agents to replace traditionally used chemical agents and antibiotics [22,23]. In addition, exposure to sub-MIC concentrations of some traditional antibiotics and disinfectants can act as an environmental signal triggering biofilm formation [24,25], supporting the research on new antimicrobial compounds. MIC values for ICs against *S. aureus* strains showed in this investigation (Table 1) were in agreement with previous studies showing the strong inhibitory activity of carvacrol, citral, and (+)-limonene against several strains of *S. aureus* and other bacterial species [21,26,27].

The study of the evolution of the biofilm mass production revealed that highly diverse patterns of adhesion and biofilm formation exist among the different assayed *S. aureus* strains (Figure 1). We found no correlation between each strain’s ability to form a biofilm and its original source of isolation and its susceptibility to methicillin. Yet, we detected that biofilm mass production is directly related to the incubation time interval, which varies from strain to strain. Therefore, incubation time should be considered when comparing biofilm formation ability among strains. For instance, the strains SC-01 and Newman exhibited increasing and decreasing biofilm thickness over 40 h of incubation period (Figure 1A,D). Thus, after 24 h of incubation, which is the usual time to evaluate antimicrobial activity of EOs [16,17,26,28], UAMS-1 was a better biofilm forming strain than USA300 and Newman (Figure 1). However, after 32 h of incubation, USA300 strain showed a thicker biofilm than Newman and UAMS-1 strains which is consistent with what has been previously reported [3,29,30].

Addition of NIC of ICs to growth medium inhibited biofilm mass production by *S. aureus* strains. Although most studies agree about the inhibitory effect of EOs and ICs on the biofilm formation [31,32,33,34,35,36,37,38,39], different authors have detected an enhancement of the biofilm production by these compounds under some conditions [40,41,42]. As described for biofilm formation in absence of ICs, the inhibitory effect of these compounds on biofilm mass production varied as a function of the time point tested. The biofilm formation is a dynamic and cyclical process that involves two initial steps; an initial attachment and a subsequent maturation phase. A final dispersal phase occurs when the biofilm reaches a nutrient-deprived critical mass or as a response to changing environmental conditions, which causes the detachment of bacteria from the outermost layers of the biofilm and become planktonic single individuals [2,18]. Therefore, dependence of the incubation period in the inhibition of biofilm mass production could be attributable to a possible delay in the cycles of biofilm assembly-disassembly caused by antimicrobial effect of ICs, emphasizing the importance of measuring the anti-biofilm properties of any compound of interest at different time points during the process of biofilm formation. Although the representation of the stages of biofilm development has been applied to the identification of particularities in mutants or the differentiation of strains [43,44], to the best of our knowledge, most studies describing the effect of EOs or ICs on the biofilm development only compare the biofilm produced after a certain period of incubation, usually 24 h [16,17,26,28]. Comparison of the area under the curve of biofilm for biofilm mass production in absence or presence of ICs (Table 2) provided us with a useful parameter to describe inhibitory properties of these compounds in biofilm formation. This parameter revealed carvacrol as a potent inhibitor of biofilm mass production by all *S. aureus* strains tested.

Although resistance to ICs (MIC values) was similar for the planktonic cells of all the strains (Table 1), inhibition of biofilm mass production varied as a function of the assayed *S. aureus* strain (Figure 1). This differential behaviour could indicate that different or specific mechanisms of biofilm inhibition (other than those related to the delay in the initial attachment) are present depending on the *S. aureus* strain and on the antimicrobial compound. Difference between the planktonic cell concentration of *S. aureus* Newman in the absence and in the presence of the NICs of the ICs after 8 h of incubation (Figure 3) could account for the difference observed in the biofilm production at this time point (Figure 1D) by delaying the initial attachment and consequently the biofilm mass production. In this regard, it is known that the first step of the biofilm development (the initial bacterial adhesion to the surface) is conditioned by the growth stage of the bacterial cells [11]. On the other hand, high cell density triggers *quorum sensing* response, based on cell-to-cell communication that regulates genes involved in biofilm maturation and maintenance [2,23]. *Quorum sensing* response is activated when the concentration of autoinducers (small molecules secreted by bacteria) exceeds a requisite threshold. Therefore, lower initial bacterial counts would delay biofilm mass production [9]. Moreover, these ICs can damage cell envelopes [20,21] which could result in a diminished ability to attach to the surface and form biofilms, as previously suggested by Kerekes *et al.* [17]. Likewise, inhibitory effects of ICs on planktonic cells could also explain the delay in biofilm formation by the other *S. aureus* strains. However, while biofilm formation by *S. aureus* Newman in presence of citral and (+)-limonene was only delayed, growth of the other strains in presence of ICs also reduced the highest biofilm mass (Table 2). Therefore, in contrast to the results obtained with Newman strain, the effect of ICs on inhibiting biofilm formation by SC-01, USA300 and UAMS-1 strains would not only be due to the inhibitory effect of ICs on planktonic cells, indicating a different mechanism of action by ICs for these strains and Newman strain.

The control of *S. aureus* in clinical settings has been traditionally performed by increasing concentrations of conventional antibiotics, such as β-lactams or glycopeptides. Unfortunately, the uncontrolled use of penicillins, like methicillin, to treat patients and livestock contributed in emergence of MRSA [45]. MRSA infections show a mortality rate of 20%, and are the leading cause of death by a single infectious agent in the USA, high above HIV [46]. In addition to this, a subset of CA-MRSA strains has emerged, which are no longer restricted to patients from hospitals or immuno-compromised high-risk citizens but in fact have the ability to cause severe and pandemic infections in healthy individuals [47]. The reduced number of effective antimicrobial treatments that are available to eradicate multi-drug resistant pathogenic bacteria could lead to recurrent microbial contaminations in food industry, and to hard-to-treat infections frequently related to hospitals. In food processing, EOs and ICs are of special interest since their natural origin meets consumers’ current reluctance towards chemically-synthesized antimicrobials [48]. In addition to their use as sanitizers and disinfectants of food equipment and environments, food packaging materials containing antimicrobial compounds have gained practical importance in the control of surface contamination [49].

The potential of sub-MIC concentrations of carvacrol, citral, and (+)-limonene as inhibitors of the biofilm formation in multi-drug resistant strains, such as the CA-MRSA strain SC-01 (Figure 2), whose biofilms can develop in the presence of many conventional antibiotics [50], should be further considered as anti-biofilm compounds. Finally, another important reason that supports the use of ICs in food and clinical environments is the evidence that, unlike conventional antibiotics, these compounds would effectively kill bacteria while applying a less selective pressure for the development of resistance [51].

## 3. Experimental Section

### 3.1. Microorganisms and Growth Conditions

The four bacterial strains of *S. aureus* used in this study (Newman, UAMS-1, USA300, and SC-01) were obtained from the Kolter laboratory (Harvard Medical School, Boston, MA, USA). Newman strain is an antibiotic sensitive (MSSA, from methicillin-sensitive *S. aureus*) isolate from an osteomyelitis patient (Royal South Wants Hospital, Southampton, UK) [52]; UAMS-1 is another MSSA osteomyelitis isolate from the University of Arkansas Medical School (Little Rock, AR, USA) [53]; USA300 is a CA-MRSA outbreak strain (USA) [45]; and SC-01 is a CA-MRSA isolate from a war veteran’s hip wound (Sepulveda Veterans Administration Medical Center, Sepulveda, CA, USA) [54]. All of them were clinical isolates, USA300 strain being a major source of community-associated outbreaks of *S. aureus* in America and Europe [55].

During this investigation, the strains were kept frozen at −80 °C in cryovials. Broth subcultures were prepared by inoculating, with one single colony from a plate, a test tube containing 5 mL of sterile Tryptic Soy Broth (TSB) (Biolife, Milan, Italy). After inoculation, the tubes were incubated overnight at 37 °C in aerobic conditions (Selecta, mod Incudigit, Barcelona, Spain) to obtain bacterial subcultures. With these subcultures, tubes containing 5 mL of TSB were inoculated to a final concentration of 10^7^ CFU/mL. These bacterial cultures were incubated under agitation (130 rpm; Selecta, mod. Rotabit) at 37 °C for 12 ± 2 h so that the stationary growth phase was reached.

### 3.2. Procedure for Biofilm Formation

For *S. aureus* biofilm formation, 0.01 mL of a bacterial culture and 0.5 mL of the growth medium were inoculated in selected wells in polystyrene 24-well plates (Nunclon Delta Surface, Thermo Fisher Scientific, Roskilde, Denmark), attaining an initial concentration of 10^7^ CFU/mL. The growth medium was composed by TSB + Glucose 0.5% (VWR, Leuven, Belgium) + NaCl 3% (Panreac, Barcelona, Spain). The plates were incubated inside individual plastic bags at 37 °C in static conditions for different incubation times. Biofilms formed by *S. aureus* were stained with crystal violet (0.1%) (Panreac) for better visualization and quantification of the biomass according to O’Toole and Kolter [56]. Briefly, the supernatant containing the planktonic cells was extracted from each well, and the plates were rinsed with distilled water to remove non-attached cells and left to air-dry for about 2 h. Next, each well was filled with 300 µL/L of crystal violet solution and incubated at room temperature (20–25 °C) for 30 min. The crystal violet solution was removed, and the wells were further washed with distilled water. To quantify biofilm formation the crystal violet stain was solubilized in 33% acetic acid (Panreac) [57] and absorbance measured at 595 nm using a microplate reader (Genios, Tecan, Männedorf, Switzerland). This technique measures total biomass (cells and extracellular matrix) [58], and therefore is suitable to determine biofilm removal or inhibition.

### 3.3. Determination of Minimum Inhibitory Concentration (MIC) and Quantification of the Effect of Sub-MIC Concentrations of EOs and ICs

For the determination of the MIC of planktonic cells 24-well plates were prepared as previously described. Growth medium had been previously added with 10, 50, 100, 200, 500, 1000, 2000, 5000, or 10,000 µL/L of carvacrol (PubChem CID: 10364), citral (PubChem CID: 638011; mixture of two isomers: geranial and neral), or (+)-limonene (PubChem CID: 440917), and plates were incubated at 37 °C for 24 h. Carvacrol (≥98%), citral (95%), and (+)-limonene (97%) were purchased from Sigma Aldrich (Sigma-Aldrich Chemie, Steinheim, Germany). Positive controls (with bacterial culture but no compounds added) and negative controls (with compounds but no bacterial culture added) were also included. MIC was noted as the lowest compound concentration at which the optical absorbance at 595 nm of the content of each well was equal or lower than that of their negative controls, and the non-inhibitory concentration (NIC) was determined as its immediately lower concentration.

The same procedure for different incubation times was followed for the biofilm formation assay throughout time in the presence of 1- or 0.1-fold NIC of carvacrol, citral, or (+)-limonene. Positive controls (with bacterial culture but no compounds added) and negative controls (with compounds but no bacterial culture added) were also included. The assays were performed in duplicate in three independent experiments.

### 3.4. Quantification of Planktonic Cells in Biofilm-Growing Cultures

The supernatant obtained from each well was homogenized (Genios 3, Ika, Königswinter, Germany) and adequately diluted in 0.1% (*w*/*v*) peptone water (Biolife, Milan, Italy). Next, 0.02-mL aliquots of each sample were inoculated in plates containing TSA (Biolife) using the spread plate technique. Plates were incubated at 37 °C for 24 h before bacterial counts (CFU/mL). The assays were performed in duplicate in three independent experiments.

### 3.5. Data Analyses

All the analyses were performed with PRISM software (GraphPad Software, Inc., San Diego, CA, USA). ANOVA tests with Bonferroni’s multiple comparison Post-test were performed to test statistically significant differences among several groups (α = 0.05). The error bars in the figures indicate the mean ± standard deviations from the obtained data.

PRISM software was also used to obtain the measurement of the cumulative absorbance values for each strain and compound throughout the total incubation time (40 h). These cumulative absorbance values were computed as the area under the curve (AUC) [59] following the trapezoid rule, where the total area is the sum of all rectangular trapezoids, each defined by two adjacent absorbance values with respect to the ground (in the y axis) and the time between those measurements (in the x axis). Therefore, the formula used was:

(1) $$\text{AUC =}\overset{\text{n-1}}{\underset{\text{i = 1}}{\sum }}\frac{x_{i}\cdot (y_{i}+y_{i\text{i+1}})}{2}$$

with *x_i_* denoting the time between measurements, *y_i_* denoting the absorbance value for each measurement, and *n* being the total number of measurements. The AUC values were determined for each individual experiment as a mean of the two replicates performed per experiment.

The AUC values were used to express the percentages of decrease in the cumulative absorbance values caused by the incorporation of each compound in the growth medium. For each strain and compound, the percentage of decrease was calculated for each individual experiment using the formula:

(2) $$\text{Percentage of decrease =}\frac{{\mathrm{AUC}}_{\text{control}}-{\text{AUC}}_{\text{compound}}}{{\text{AUC}}_{\text{control}}}\;\times 100$$

where AUC_control_ and AUC_compound_ were the cumulative absorbance values in the absence of added compounds or in the presence of each one of them, respectively. The mean and standard deviation of three individual experiments were also calculated using PRISM software.

## 4. Conclusions

In brief, we have shown the importance of studying the evolution of the biofilm development throughout time when describing the effect of ICs on the biofilm formation by each bacterial strain. Due to the cyclic behavior inherent to the biofilm development, the measured biofilm mass fluctuates in response to the disassembly and reforming on the biofilm. The four clinical *S. aureus* strains showed different patterns of biofilm development, and also exhibited different sensitivity to the ICs carvacrol, citral, and (+)-limonene. Overall, the three ICs acted similarly, although analysis of the cumulative biofilm production highlighted carvacrol as the best inhibitor of biofilm mass production, decreasing more than 40% of biofilm mass production for any strain. Interestingly, our ICs were capable of reducing biofilm mass of SC-01 strain in 65%–85% when incorporating the NIC of any IC (100, 200, and 2000 µL/L of carvacrol, citral, and (+)-limonene, respectively) in the growth medium (after 16 h of incubation). These compounds also inhibited biofilm mass production by SC-01 at concentrations 10 times lower than the NIC. This is particularly important in CA-MRSA strains, which are able to grow and form biofilm in the presence of many different conventional antibiotics.

The mechanism(s) by which ICs modify *S. aureus’* pattern of biofilm development are still unknown. The absence or delay in the first peak of the biofilm development in presence of ICs, along with the initial partial inactivation of planktonic cells in the strain Newman, suggested that the delay in the attachment of cells would probably be one of the factors implied in the effectiveness of the tested ICs.

The anti-biofilm properties of our molecules suggest that they may have a great potential to fight against bacterial biofilms in the food industry. Furthermore, ICs could be used to treat biofilm-associated infections caused by multi-drug resistant pathogenic bacteria.
